# Patient-reported outcomes following stereotactic body radiation therapy for clinically localized prostate cancer

**DOI:** 10.1186/1748-717X-9-52

**Published:** 2014-02-11

**Authors:** Onita Bhattasali, Leonard N Chen, Jennifer Woo, Jee-Won Park, Joy S Kim, Rudy Moures, Thomas Yung, Siyuan Lei, Brian T Collins, Keith Kowalczyk, Simeng Suy, Anatoly Dritschilo, John H Lynch, Sean P Collins

**Affiliations:** 1 Department of Radiation Medicine, Georgetown University Hospital, 3800 Reservoir Road, N.W,, Washington, D.C 20007, USA; 2 Department of Urology, Georgetown University Hospital, 3800 Reservoir Road, N.W, Washington, D.C 2007, USA

**Keywords:** Prostate cancer, SBRT, CyberKnife, EPIC, Bother, Function, Late symptom flare

## Abstract

**Background:**

Stereotactic body radiation therapy (SBRT) delivers high doses of radiation to the prostate while minimizing radiation to adjacent normal tissues. Large fraction sizes may increase the risk of functional decrements. Treatment-related bother may be more important to a patient than treatment-related dysfunction. This study reports on patient-reported outcomes following SBRT for clinically localized prostate cancer.

**Methods:**

Between August 2007 and July 2011, 228 consecutive hormone-naïve patients with clinically localized prostate cancer were treated with 35–36.25 Gy SBRT delivered using the CyberKnife Radiosurgical System (Accuray) in 5 fractions. Quality of life was assessed using the American Urological Association Symptom Score (AUA) and the Expanded Prostate Cancer Index Composite (EPIC)-26. Urinary symptom flare was defined as an AUA score 15 or more with an increase of 5 or more points above baseline 6 months after treatment.

**Results:**

228 patients (88 low-, 126 intermediate- and 14 high-risk) at a median age of 69 (44–90) years received SBRT with a minimum follow-up of 24 months. EPIC urinary and bowel summary scores declined transiently at 1 month and experienced a second, more protracted decline between 9 months and 18 months before returning to near baseline 2 years post-SBRT. 14.5% of patients experienced late urinary symptom flare following treatment. Patients who experienced urinary symptom flare had poorer bowel quality of life following SBRT. EPIC scores for urinary bother declined transiently, first at 1 month and again at 12 months, before approaching pre-treatment scores by 2 years. Bowel bother showed a similar pattern, but the second decline was smaller and lasted 9 months to 18 months. EPIC sexual summary and bother scores progressively declined over the 2 years following SBRT without recovery.

**Conclusions:**

In the first 2 years, the impact of SBRT on urination and defecation was minimal. Transient late increases in urinary and bowel dysfunction and bother were observed. However, urinary and bowel function and bother recovered to near baseline by 2 years post-SBRT. Sexual dysfunction and bother steadily increased following treatment without recovery. SBRT for clinically localized prostate cancer was well tolerated with treatment-related dysfunction and bother comparable to conventionally fractionated radiation therapy or brachytherapy.

## Background

Stereotactic body radiation therapy (SBRT) is establishing itself as a new modality for the treatment of clinically localized prostate cancer [1,2]. SBRT delivers high doses of radiation to target volumes with precision while minimizing radiation exposure to adjacent healthy tissues [3,4]. With SBRT, biochemical disease-free survival is high [5] while toxicity has been comparable to conventionally fractionated radiation therapy despite higher doses per fraction [5-8]. Presently, there is limited data suggesting that any particular treatment for prostate cancer has superior outcomes compared to the others [9]. As a result, the choice of intervention is guided by the treatment’s side effect profile and the patient’s subsequent health-related quality of life (HRQOL) [10].

Commonly employed prostate cancer-specific quality of life (QOL) questionnaires contain questions that assess both function and bother (the annoyance that patients experience due to functional decrements) [11,12]. Several studies have assessed QOL outcomes following SBRT for clinically localized prostate cancer [2,5,13]. These studies have primarily focused on functional decrements following treatment. Bother, a subjective measure of QOL, may be more important to an individual patient than treatment-related dysfunction. While function and bother share an association, it varies across specific domains [14]. Even within a given domain, function and bother may vary over time [10-12]. With time, patients may come to accept functional deficits and become less bothered by them [11,12,15,16]. Bother may be more affected by the patient’s expectations prior to treatment rather than the severity of the functional decrement [12,17,18].

Limited data to date is available on patient-reported outcomes following SBRT. Further knowledge in this area would facilitate better communication between patients and physicians when deciding on the appropriate management route. The objective of this study is to report the urinary, bowel, and sexual QOL outcomes following SBRT in patients with clinically localized prostate cancer.

## Methods

### Patient selection

Patients eligible for study inclusion had histologically-confirmed adenocarcinoma of the prostate treated per our institutional protocol. Patients who received ADT were excluded from this study due to its known adverse effects on patient-reported outcomes [19]. Georgetown University Institutional Review Board approval was obtained for retrospective review of data that was prospectively collected in our institutional database.

### SBRT treatment planning and delivery

SBRT treatment planning and delivery was performed as previously described [4,7]. Gold fiducials were placed into the prostate using ultrasound guidance. Fused thin cut CT images and high-resolution MR images were used for treatment planning. The clinical target volume (CTV) included the prostate and proximal seminal vesicles. The planning target volume (PTV) included a 5 mm anterolateral expansion and a 3 mm posterior expansion around the CTV. A prescription dose of 35–36.25 Gy was delivered to the PTV in 5 fractions of 7–7.25 Gy over 1–2 weeks. The bladder, membranous urethra, rectum, and penile bulb were contoured and evaluated with dose-volume histogram analysis during treatment planning. Target position was confirmed multiple times during each treatment with a minimum of 3 properly-placed fiducials [4].

### Follow-up and statistical analysis

Patients completed the Expanded Prostate Cancer Index Composite (EPIC)-26 [20], American Urological Association Symptom Index (AUA) [21], and Sexual Health Inventory for Men (SHIM) questionnaires [22] before treatment and during routine follow-up visits 1 month after the completion of SBRT, every 3 months for the first year, and then every 6 months for the second year. The EPIC-26 is a validated tool that measures urinary, bowel, and sexual function and bother [20]. To statistically compare changes between time points, the levels of responses were assigned a score, and the significance of the mean changes in the scores was assessed by paired *t*-test. Responses to the EPIC-26 questionnaire were grouped by physiologic domains and assigned numerical scores. The multi-item scale scores were transformed linearly to a 0–100 scale as recommended in the scoring instructions for the EPIC-26. Lower numbers corresponded to worsening function and increased bother. For the overall urinary, bowel, and sexual bother questions (Questions 5, 7, and 12), the responses were grouped into 3 clinically relevant categories (no problem, very-small to small problem, and moderate to big problem). Wilcoxon Signed-Rank Test and chi-square analysis were used to assess differences in QOL scores in comparison to baseline. Paired *t*-test was used to assess the significance of the change in scores. The minimally important difference (MID) to assess for clinically significant change in HRQOL from baseline was set as half a standard deviation (SD) [23]. As previously reported, late urinary symptom flare was defined as an increase of ≥ 5 points above baseline with a degree of severity in the moderate to severe range (AUA score ≥ 15) [7,24]. Statistical analysis was limited to time points with a ≥ 80% response rate to limit the effect of attrition bias.

## Results

From August 2007 to July 2011, 228 hormone-naïve patients with clinically localized prostate adenocarcinoma were treated per our institutional SBRT monotherapy protocol. The patients were followed for a minimum of 24 months following SBRT. The median patient age was 69 (44–90) years old (Table 1). 58.8% patients were white, and 35.5% were black. 75.4% patients had partners, and 47.4% patients were employed. 38.6% patients were low-risk, 55.3% patients were intermediate-risk, and 6.1% patients were high-risk. The median prostate volume was 37.3 (11.6-138.7) cc. 48% of patients had moderate to severe lower urinary tract symptoms prior to treatment (baseline AUA ≥ 8) with a median baseline AUA of 7 (Table 2). The median pre-treatment testosterone was 11 (3.99-39.87) nmol/L. 77.2% of patients had erectile dysfunction prior to treatment (baseline SHIM ≤ 21) with a median baseline SHIM of 16 (Table 2). 38.1% of patients utilized sexual aids prior to SBRT.

**Table 1:** Patient characteristics

		**Patients (N = 228)**
**Age (y/o)**	(Median = 69)	
	Age ≤ 60	11.0%
	60 < Ages ≤ 70	45.6%
	Age > 70	43.4%
**Race**		
	White	58.8%
	Black	35.5%
	Other	5.7%
**Partner status**		
	Yes	75.40%
	No	24.60%
**Employment status**		
	Yes	47.40%
	No	52.60%
**Median pre-treatment PSA (ng/mL)**		6.1 (1.3-32.5) ng/dL
**Median pre-treatment Testosterone (nmol/L)**		11 (3.99-39.87) nmol/L
**Risk groups (D’Amico’s)**		
	Low risk	38.6%
	Intermediate risk	55.3%
	High risk	6.1%
**Sexual aid**		
	None	61.9%
	Yes (Any)	38.1%
**SBRT dose**		
	36.25 Gy	84.2%
	35 Gy	13.2%
	Other	2.6%

**Table 2:** Pre-treatment Quality of Life (QOL) scores

	**% Patients (n=228)**		
**Baseline AUA score**			
0-7 (Mild)	52.0%		
8-19 (Moderate)	44.5%		
≥ 20 (Severe)	3.5%		
			
**Baseline SHIM score**			
22-25 (No ED)	23.8%		
17-21 (Mild ED)	22.9%		
12-16 (Mild-Moderate ED)	13.9%		
8-11 (Moderate ED)	5.4%		
< 8 (Severe ED)	34.1%		
			
**Baseline EPIC-26 summary score**	Mean	SD	MID
Urinary domain	89.6	10.92	5.5
Bowel domain	95.1	8.55	4.3
Sexual domain	56.3	28.92	14.5
			
**Baseline EPIC-26 bother score**	Mean	SD	MID
Urinary domain	80.2	24.45	12.2
Bowel domain	91.4	18.49	9.2
Sexual domain	65.9	34.49	17.2

*Abbreviation: AUA *American Urological Association symptom scores, *SHIM* Sexual Health Inventory for Men, *EPIC* Expanded Prostate Cancer Index Composite, *SD* Standard Deviation, *MID* Minimally Important Difference.

84% of patients were treated with 36.25 Gy in five 7.25 Gy fractions (Table 1). The median follow-up was 3.8 years. The median pre-treatment PSA of 6.1 ng/ml declined by two years post-treatment to a median PSA of 0.49 ng/ml. There were 6 biochemical failures, occurring in 1 low-risk patient, 3 intermediate-risk patients, and 2 high-risk patients. The overall 2-year actuarial biochemical relapse-free survival was 97.2%. No patients received androgen deprivation therapy at any time during the first 2 years following SBRT.

**Figure 1:**
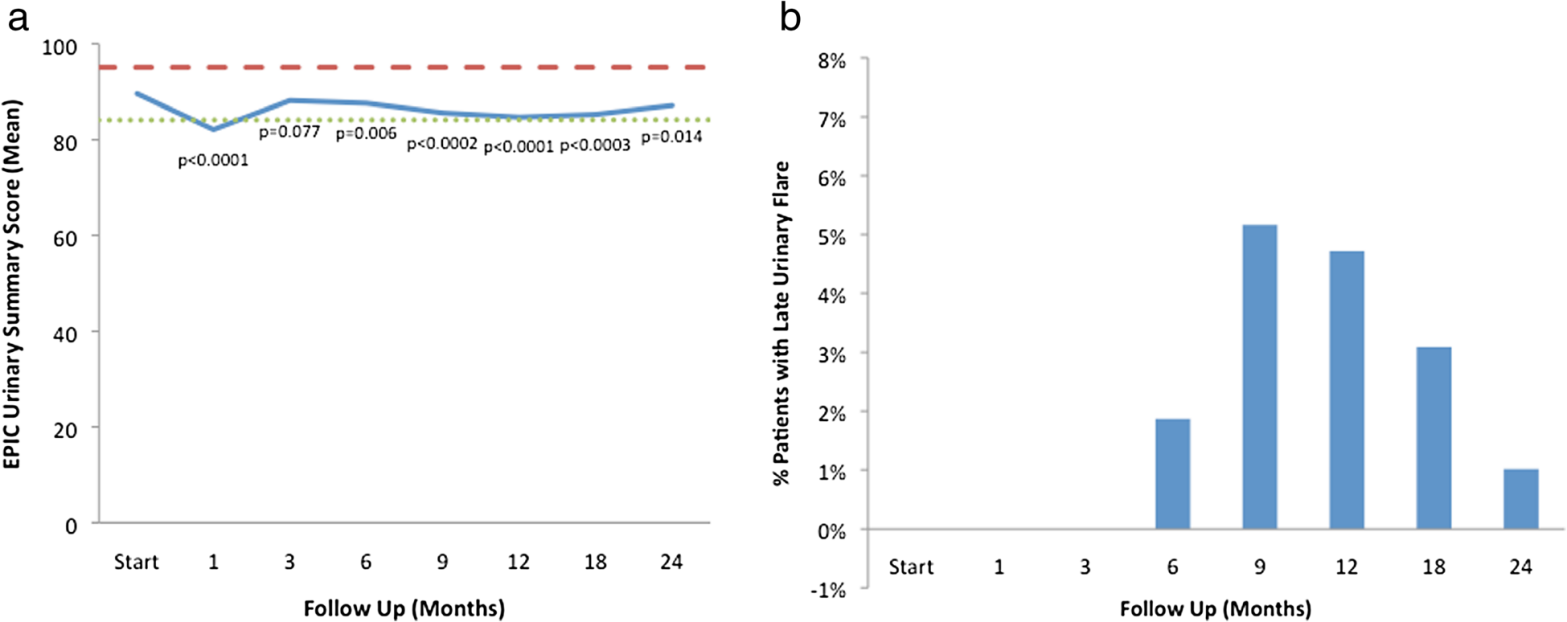
**Urinary function. (a)** EPIC urinary summary domain scores at baseline and following SBRT for prostate cancer. Thresholds for clinically significant changes in scores (½ standard deviation above and below the baseline) are marked with dashed lines. EPIC scores range from 0–100 with higher values representing a more favorable health-related QOL. **(b)** Percentage of patients with urinary symptom flare at each follow-up.

Baseline EPIC summary scores are shown in Table 2 and mean changes in EPIC summary scores from baseline to 2 years of follow-up are shown in Table 3. The EPIC urinary summary score declined transiently at 1 month post-SBRT (mean change from baseline, -7.6) (Table 3) and returned to near baseline by 3 months post-SBRT (mean change from baseline, -1.5) (Table 3, Figure 1a). This acute decline was both statistically (*p* < 0.0001) and clinically significant (MID = 5.5). A second late, protracted decline occurred between 9 months and 18 months (mean change from baseline at 12 months, -5.1) (Table 3). Transient late urinary symptom flare (≥ 6 months after completing treatment) occurred in 14.5% of the patients (Figure 1b) [7]. The median flare magnitude was 14 (5–30) and the median time to flare was 12 (6–24) months. The EPIC urinary summary score returned to near baseline by 2 years post-SBRT (mean change from baseline, -2.6) (Table 3, Figure 1a).

**Table 3:** Patient-reported quality of life domain summary scores in three EPIC-26 domains

	**1 month post-treatment (n = 219)**			**3 month post-treatment (n = 215)**			**12 month post-treatment (n = 210)**			**24 month post-treatment (n = 197)**		
**Domain**	**Mean score change from baseline**	**SD**	***P***	**Mean score change from baseline**	**SD**	***P***	**Mean score change from baseline**	**SD**	***P***	**Mean score change from baseline**	**SD**	***P***
Urinary summary	-7.6	13.2	<0.0001	-1.5	11.9	0.0769	-5.1	14.9	<0.0001	-2.6	14.8	0.0135
Bowel summary	-10.1	17.7	<0.0001	-3.1	11.4	0.0002	-3.4	11.7	<0.0001	-1.4	9.8	0.0512
Sexual summary	-5.7	29.5	<0.0001	-4.2	30.9	0.0007	-9.2	30.4	<0.0001	-11.8	30.8	<0.0001

**Figure 2:**
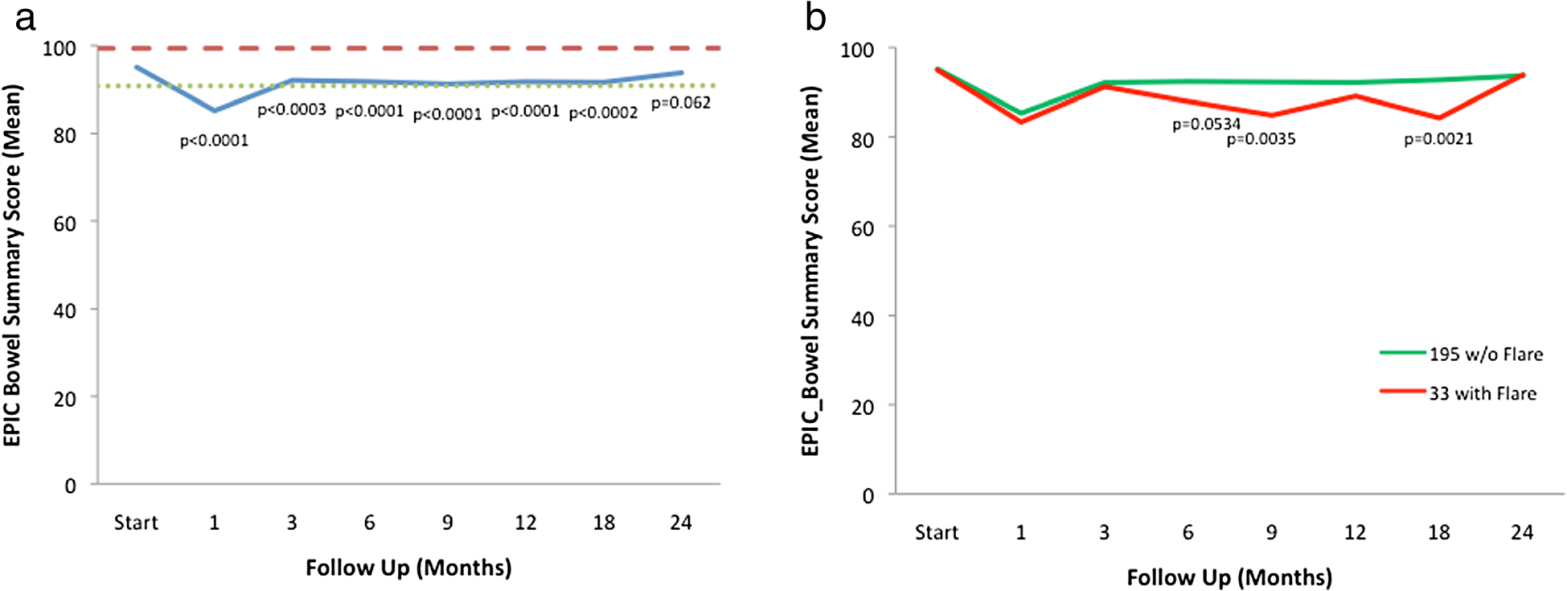
**Bowel function. (a)** EPIC bowel summary domain scores at baseline and following SBRT for prostate cancer. **(b)** EPIC bowel summary domain scores in patients with or without urinary symptom flare. Thresholds for clinically significant changes in scores (½ standard deviation above and below the baseline) are marked with dashed lines. EPIC scores range from 0–100 with higher values representing a more favorable health-related QOL.

Likewise, the EPIC bowel summary score declined transiently at 1 month (mean change, -10.1) (Table 3) and experienced a second, more protracted decline between 9 months and 18 months (mean change from baseline at 12 months, -3.4). Bowel declines at 1 month and 12 months were statistically significant (*p* < 0.0001); however, only the change at 1 month met the threshold for clinically significant change (MID = 4.3) (Figure 2a). Transient late declines in the EPIC bowel summary domain were more common in patients who experienced late urinary symptom flare (Figure 2b). The EPIC bowel summary score returned to near baseline at 2 years post-SBRT (mean change from baseline, -1.4) (Table 3, Figure 2a).

**Figure 3:**
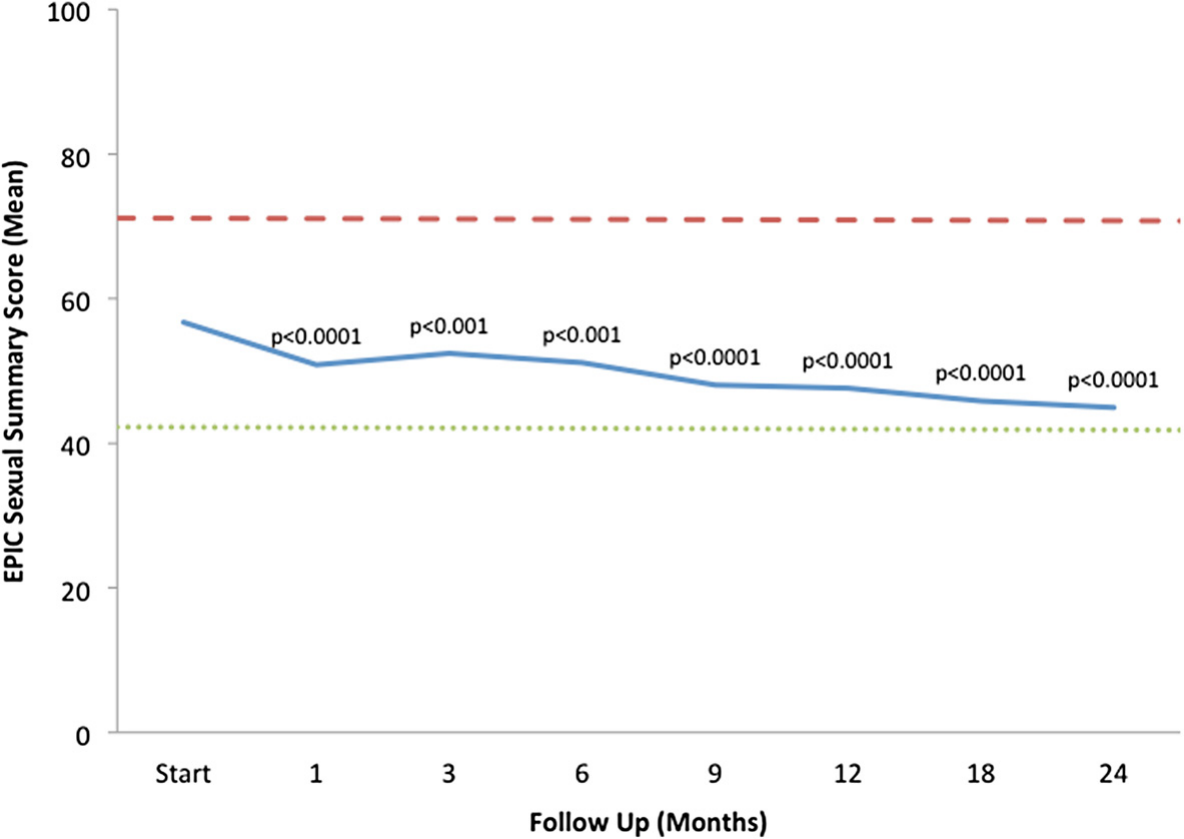
**Sexual function.** EPIC sexual summary domain scores at baseline and following SBRT for prostate cancer. Thresholds for clinically significant changes in scores (½ standard deviation above and below the baseline) are marked with dashed lines. EPIC scores range from 0–100 with higher values representing a more favorable health-related QOL.

The EPIC sexual summary score progressively declined over 24 months (Figure 3). Sexual declines were statistically significant (*p* < 0.001) at all follow-ups, but the results were not found to be clinically significant due to the high variation in baseline sexual function [25] and consequent EPIC sexual summary score in the study population (MID = 14.5) (Table 2).

**Figure 4:**
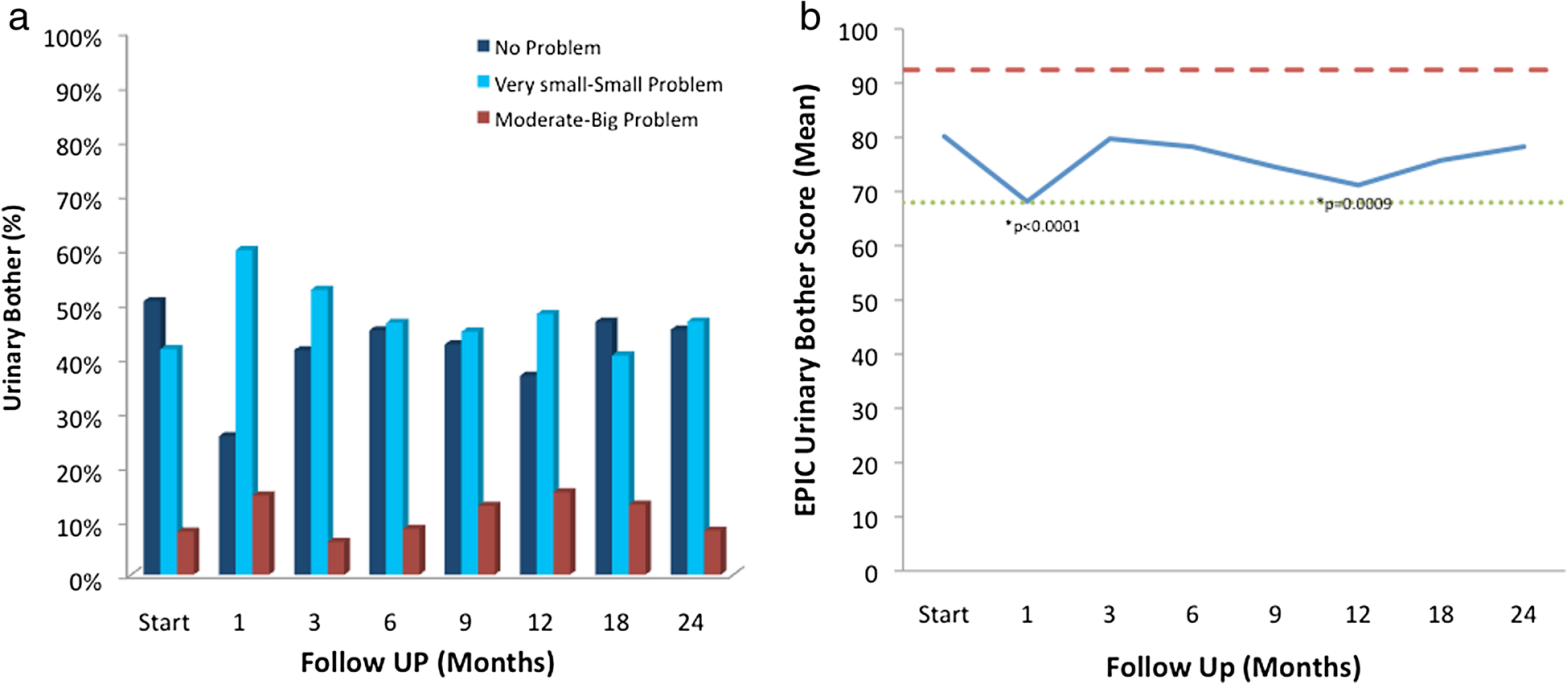
**EPIC urinary bother at baseline and following SBRT for prostate cancer. (a)** Urinary bother was stratified to three levels of bother: no problem, very small-small problem, and moderate-big problem. **(b)** Average overall urinary bother scores (Question 5 of the EPIC-26). Thresholds for clinically significant changes in scores (½ standard deviation above and below the baseline) are marked with dashed lines. EPIC scores range from 0–100 with higher values representing a more favorable health-related QOL.

Treatment-related bother may be more important to an individual patient than treatment-related dysfunction. At baseline, nearly half (49.6%) of our cohort reported some level of annoyance due to urinary symptoms with 8.0% of the patients feeling that urination was a moderate to big problem (Table 4, Figure 4a). Baseline EPIC bother scores are shown in Table 2 and mean changes in EPIC bother scores from baseline to 2 years of follow-up are shown in Table 5. The mean EPIC urinary bother score was 80.2 at baseline (Table 2). Urinary bother increased following treatment with the mean score decreasing to 68.2 at 1 month post-treatment (mean change, -12) (*p* < 0.0001) (Table 5, Figure 4b). However, only 14.6% of patients felt that urination was a moderate to big problem at 1 month following treatment (Table 4, Figure 4a). Although urinary bother declined quickly, a second late increase in urinary bother was observed with the mean urinary bother score decreasing to 71.2 at 12 months (mean change from baseline, -9) (p = 0.0009) (Figure 4b). Only the first decline approached the threshold for clinically significant change (MID =12.2). However, 15.2% of patients felt that urination was a moderate to big problem at 12 months following treatment (Table 4, Figure 4a). By two years following SBRT, urinary bother returned to near baseline with a urinary bother score of 78.3 (mean change from baseline, -1.9) and 8.1% of patients feeling that urination was a moderate to big problem (Table 4, Figure 4a).

**Table 4:** EPIC bothers

	**Start**	**1 Mon**	**3 Mon**	**6 Mon**	**9 Mon**	**12 Mon**	**18 Mon**	**24 Mon**
**Total N**	**226**	**219**	**215**	**213**	**212**	**210**	**193**	**197**
**Urinary bother**								
**No problem**	50.4%	25.6%	41.4%	45.1%	42.5%	36.7%	46.6%	45.2%
**Very small-small problem**	41.6%	59.8%	52.6%	46.5%	44.8%	48.1%	40.4%	46.7%
**Moderate-big problem**	8.0%	14.6%	6.0%	8.5%	12.7%	15.2%	13.0%	8.1%
*p=*		<0.0001				0.0009		
**Bowel bother**								
**No problem**	77.0%	44.3%	66.5%	68.5%	63.2%	63.3%	66.1%	72.1%
**Very small-small problem**	19.5%	44.7%	30.2%	28.2%	31.6%	30.0%	29.7%	25.4%
**Moderate-big problem**	3.5%	11.0%	3.3%	3.3%	5.2%	6.7%	4.2%	2.5%
*p=*		<0.0001			0.0017	0.001	0.0408	
**Sexual bother**								
**No problem**	39.4%	37.0%	36.4%	39.0%	37.9%	40.3%	36.3%	36.2%
**Very small-small problem**	36.3%	35.2%	37.9%	31.0%	30.3%	32.2%	29.5%	29.1%
**Moderate-big problem**	24.3%	27.9%	25.7%	30.0%	31.8%	27.5%	34.2%	34.7%
*p=*							0.0022	0.0037

**Table 5:** Patient-reported quality of life bother scores in three EPIC-26 domains

	**1 month post-treatment (n = 219)**			**3 month post-treatment (n = 215)**			**12 month post-treatment (n = 210)**			**24 month post-treatment (n = 197)**		
**Domain**	**Mean score change from baseline**	**SD**	**P**	**Mean score change from baseline**	**SD**	***P***	**Mean score change from baseline**	**SD**	***P***	**Mean score change from baseline**	**SD**	***P***
Urinary bother	-12	26	<0.0001	-0.4	21.6	0.8182	-9	29.8	0.0009	-1.9	25.8	0.773
Bowel bother	-15.4	26.7	<0.0001	-3.6	20.1	0.0719	-5.7	23	0.001	-0.8	18.1	0.984
Sexual bother	-2.5	35.3	0.24	-1.1	34.3	0.563	-2.7	36.9	0.097	-7.5	37.8	0.0037

**Figure 5:**
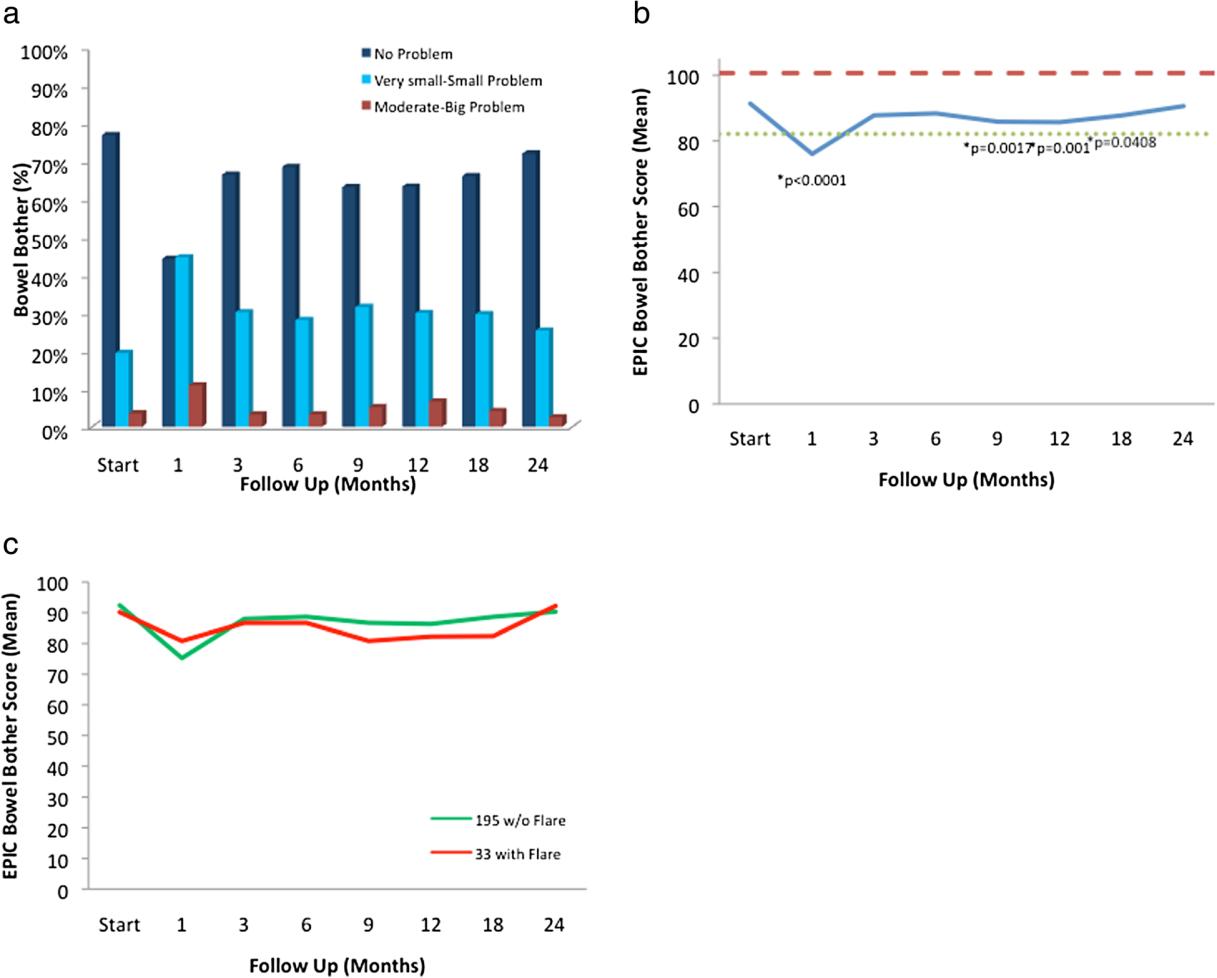
**EPIC bowel bother at baseline and following SBRT for prostate cancer. (a)** Bowel bother was stratified to three levels of bother: no problem, very small-small problem, and moderate-big problem. **(b)** Average overall bowel bother scores (Question 7 of the EPIC 26). **(c)** EPIC bowel bother scores in patients with or without urinary symptom flare. Thresholds for clinically significant changes in scores (½ standard deviation above and below the baseline) are marked with dashed lines. EPIC scores range from 0–100 with higher values representing a more favorable health-related QOL.

Bowel bother showed a similar pattern as urinary bother, but the second increase in the bowel bother score was smaller and lasted 9 months to 18 months (Table 4, Figure 5). At baseline, 23.0% of patients reported some level of annoyance due to bowel symptoms with 3.5% of patients feeling that bowel function was a moderate to big problem (Table 4, Figure 5a). The mean EPIC bowel bother score was 91.4 at baseline (Table 2). Bowel bother worsened post-treatment, and the mean score decreased to 76.0 at 1 month (mean change, -15.4) (p < 0.0001) (Table 5, Figure 5b). However, only 11% of patients felt that their bowel function was a moderate to big problem at 1 month following SBRT (Table 4, Figure 5a). Bowel bother scores worsened over a second, more protracted time period (Figure 5b). Statistical significant declines in bowel bother scores included 85.8 at 9 months (p = 0.0017), 85.8 at 12 months (mean change from baseline, -5.7) (p = 0.001) (Table 5), and 87.8 at 18 months (p = 0.041) (Figure 5b). Once again, only the first decline met the threshold for clinically significant change (MID = 9.2). Only 6.7% of patients felt that bowel symptoms were a moderate to big problem at 12 months following treatment (Table 4, Figure 5a). Once again, transient late declines in the EPIC bowel bother score were more common in patients who experienced late urinary symptom flare (Figure 5c). By 2 years following SBRT, bowel bother returned to near baseline with a bowel bother score of 90.6 (mean change from baseline, -0.8) (Table 5) and 2.5% of patients feeling bowel symptoms were a moderate to big problem (Table 4, Figure 5a).

**Figure 6:**
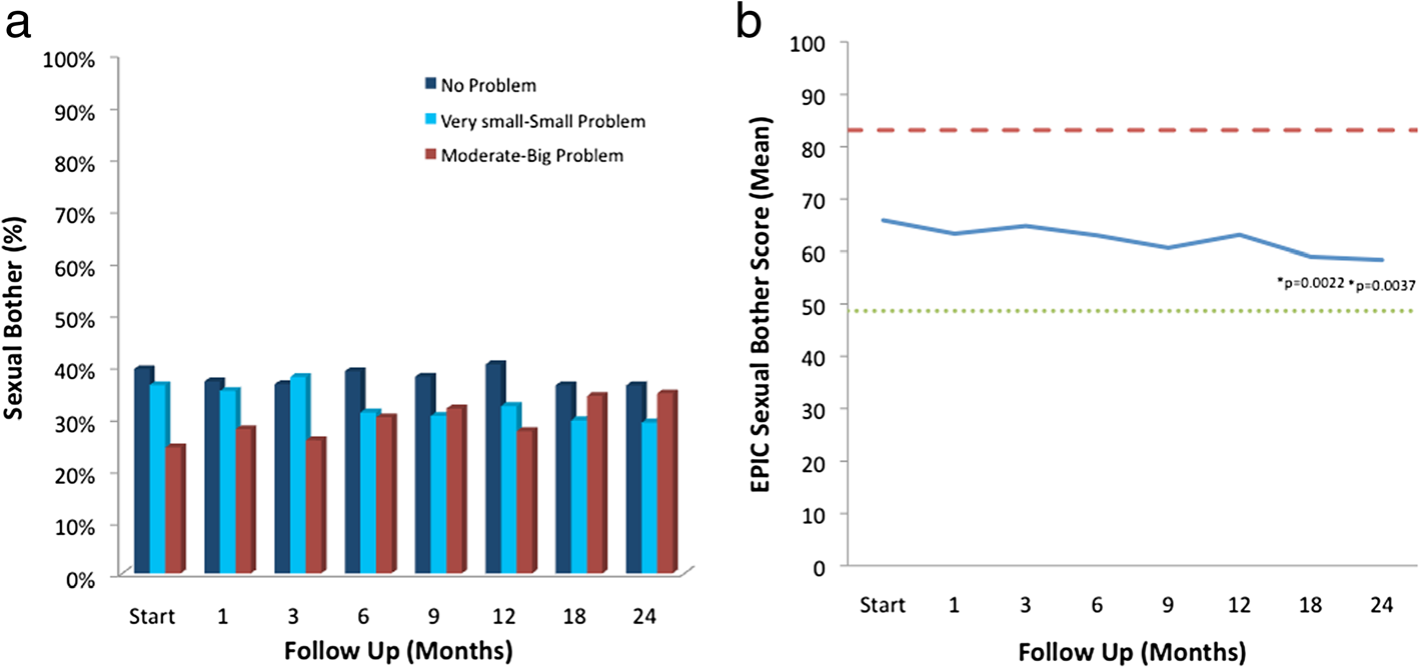
**EPIC sexual bother at baseline and following SBRT for prostate cancer. (a)** Sexual bother was stratified to three levels of bother: no problem, very small-small problem, and moderate-big problem. **(b)** Average overall sexual bother scores (Question 12 of the EPIC 26). Thresholds for clinically significant changes in scores (½ standard deviation above and below the baseline) are marked with dashed lines. EPIC scores range from 0–100 with higher values representing a more favorable health-related QOL.

Sexual bother slowly increased over the first 24 months following SBRT without recovery (Table 4, Figure 6). 60.6% of patients reported some level of sexual bother at baseline with 24.3% of the patients feeling that sex was a moderate to big problem (Table 4, Figure 6a). Worsening of sexual bother was statistically significant at 18 months (p = 0.0022) and 24 months (p = 0.0037) (Figure 6b). Mean EPIC sexual bother scores progressively declined from 65.9 at baseline to 58.4 by the end of the study (mean change from baseline, -7.5) (Table 5, Figure 6b). The results were not found to be clinically significant due to the high variation in the EPIC sexual bother score in the study population (MID = 17.2) (Table 2). 35.2% reported that sexual bother was a moderate-big problem by 24 months post-SBRT (Table 4, Figure 6a).

## Discussion

Post-treatment urinary, bowel, and sexual outcomes are important considerations in the management of clinically localized prostate cancer. Because SBRT is a newer management option for prostate cancer, there is limited data regarding outcomes after therapy. Our prior work focused on functional decrements following treatment [1,2]. Bother may be more important to an individual patient than treatment-related dysfunction. Further knowledge in this area would facilitate better communication between patients and physicians when deciding on the appropriate management route. Understanding the risk of dysfunction prior to beginning treatment may alleviate bother related to dysfunction. This study assessed patient-reported urinary, bowel, and sexual function and associated bother following SBRT.

Although SBRT delivers highly precise doses, there is unavoidable dose to the bladder neck and urethra that may cause urinary dysfunction and bother. Moderate to severe urinary bother increased following SBRT from a baseline of 8% to 15% 1 month following treatment. This compares favorably to the 30-39% of patients who report acute moderate to severe urinary bother following alternative radiation modalities [10]. As seen with conventionally fractionated external beam radiation therapy [10], this increase in urinary bother was transient and returned to baseline by 3 months post-SBRT. Similar to brachytherapy [10], a second increase in urinary bother occurred 12 months post-SBRT with 15% of patients reporting moderate to severe bother at that time point. This late bother may be associated with the phenomenon of urinary symptom flare [7,24,26,27]. Unlike brachytherapy, urinary bother returned to near baseline by 2 years post-SBRT [10,24,26,27]. Bother may have exceeded its associated dysfunction due to an unexpected increase in urinary symptoms occurring months after the expected early symptoms had already resolved. Patient counseling regarding urinary symptom flare may minimize increased urinary bother 1 year following SBRT.

Bowel bother following SBRT showed a similar pattern as urinary bother. Moderate to severe bowel bother increased following SBRT from a baseline of 3.5% to 11% 1 month following SBRT. This compares favorably to the 15-16% of patients who report acute moderate to severe bowel bother following alternative radiation modalities [10,28]. Unlike alternative modalities, this increase in bowel bother was transient and returned to near baseline by 3 months post-SBRT [10,28]. A second gradual increase in bowel bother occurred from 9 months to 18 months with approximately 7% of patients reporting moderate to severe bowel bother at 12 months. Similar to urinary bother, post-SBRT bowel bother returned to near baseline by 2 years post-SBRT.

Increased rates of bowel toxicity have been previously described in patients who experienced urinary symptom flare following brachytherapy [27]. To our knowledge, this is the first report showing an association between late urinary symptom flare and increased late bowel dysfunction and bother following SBRT. The etiology of this association is unclear but could be related to the close proximity of the involved organs [29] or due to the inherent increased radiation sensitivity in patients who experience urinary symptom flare [27,30].

Sexual bother demonstrated the poorest outcomes among all domains. As seen by others [25], a large proportion of our patients had erectile dysfunction prior to treatment. Moderate to severe sexual bother progressively increased from 24% at baseline to 35% by 2 years post-SBRT. While a small portion of the decline may be attributed to increasing age and the natural progression of erectile dysfunction, it is unlikely that age would be the sole reason for this decline. Multiple sexual aids exist to improve sexual function and may alleviate bother [31]. Patients may benefit from education regarding these available and effective enhancement tools.

Although the results of this study are promising, there remains uncertainty and a lack of data regarding longer-term toxicity from SBRT. Given the resolution of urinary and bowel toxicity by 2 years, the likelihood of a resurgence of symptoms remains low. Definitive conclusions will require the analysis of longer-term follow-up.

## Conclusions

SBRT for clinically localized prostate cancer was well tolerated with treatment-related function and bother comparable to alternative treatments [10,32]. Patients reported urinary and bowel problems at 1 month that returned to baseline by 24 months. Bothersome late urinary and bowel symptoms presented transiently between 9–18 months post-SBRT but resolved by 2 years. Sexual function and bother decreased progressively over 24 months. Educating patients regarding both the acute and delayed effects of SBRT and addressing expectations prior to treatment may decrease bother.

### Consent

This retrospective review of prospectively collected data was approved by the Georgetown University Institutional Review Board.

## Abbreviations

ADT: Androgen deprivation therapy; CTV: Clinical target volume; DVH: Dose-volume histogram; EPIC: Expanded Prostate Index Composite; GTV: Gross target volume; HRQOL: Health-related quality of life; MID: Minimally important difference; NVBs: Neurovascular bundles; PB: Penile bulb; PTV: Planning target volume; QOL: Quality of life; EBRT: External beam radiation therapy; SBRT: Stereotactic body radiation therapy.

## Competing interests

SP Collins and BT Collins serve as clinical consultants to Accuray Inc. The Department of Radiation Medicine at Georgetown University Hospital receives a grant from Accuray to support a research coordinator. The other authors declare that they have no competing interests.

## Authors’ contributions

OB and LC are lead authors who participated in manuscript drafting, table/figure creation, and manuscript revision. JW, JP, JK, RM, and TY aided in data collection and table/figure creation. SL is the dosimetrist who contributed dosimetric data and figures. SL, BC, KK, SS, AD, and JL are senior authors who aided in drafting the manuscript and manuscript revision. SC is the corresponding author who initially developed the concept, and drafted and revised the manuscript. All authors read and approved the final manuscript.

## References

[B1] King CR Freeman D Kaplan I Fuller D Bolzicco G Collins S Meier R Wang J Kupelian P Steinberg M Katz A Stereotactic body radiotherapy for localized prostate cancer: pooled analysis from a multi-institutional consortium of prospective phase II trials Radiother Oncol 2013 109 217 221 10.1016/j.radonc.2013.08.030 24060175

[B2] King CR Collins S Fuller D Wang PC Kupelian P Steinberg M Katz A Health-related quality of life after stereotactic body radiation therapy for localized prostate cancer: results from a multi-institutional consortium of prospective trials Int J Radiat Oncol Biol Phys 2013 87 939 945 10.1016/j.ijrobp.2013.08.019 24119836

[B3] Xie Y Djajaputra D King CR Hossain S Ma L Xing L Intrafractional motion of the prostate during hypofractionated radiotherapy Int J Radiat Oncol Biol Phys 2008 72 236 246 10.1016/j.ijrobp.2008.04.051 18722274 PMC2725181

[B4] Lei S Piel N Oermann EK Chen V Ju AW Dahal KN Hanscom HN Kim JS Yu X Zhang G Six-dimensional correction of intra-fractional prostate motion with CyberKnife stereotactic body radiation therapy Front Oncol 2011 1 48 22655248 10.3389/fonc.2011.00048 PMC3356099

[B5] Katz AJ Santoro M Diblasio F Ashley R Stereotactic body radiotherapy for localized prostate cancer: disease control and quality of life at 6 years Radiat Oncol 2013 8 118 10.1186/1748-717X-8-118 23668632 PMC3674983

[B6] Freeman DE King CR Stereotactic body radiotherapy for low-risk prostate cancer: five-year outcomes Radiat Oncol 2011 6 3 10.1186/1748-717X-6-3 21219625 PMC3022740

[B7] Chen LN Suy S Uhm S Oermann EK Ju AW Chen V Hanscom HN Laing S Kim JS Lei S Stereotactic body radiation therapy (SBRT) for clinically localized prostate cancer: the Georgetown University experience Radiat Oncol 2013 8 58 10.1186/1748-717X-8-58 23497695 PMC3610192

[B8] McBride SM Wong DS Dombrowski JJ Harkins B Tapella P Hanscom HN Collins SP Kaplan ID Hypofractionated stereotactic body radiotherapy in low-risk prostate adenocarcinoma: preliminary results of a multi-institutional phase 1 feasibility trial Cancer 2012 118 3681 3690 10.1002/cncr.26699 22170628

[B9] D’Amico AV Whittington R Malkowicz SB Schultz D Blank K Broderick GA Tomaszewski JE Renshaw AA Kaplan I Beard CJ Wein A Biochemical outcome after radical prostatectomy, external beam radiation therapy, or interstitial radiation therapy for clinically localized prostate cancer JAMA 1998 280 969 974 10.1001/jama.280.11.969 9749478

[B10] Sanda MG Dunn RL Michalski J Sandler HM Northouse L Hembroff L Lin X Greenfield TK Litwin MS Saigal CS Quality of life and satisfaction with outcome among prostate-cancer survivors N Engl J Med 2008 358 1250 1261 10.1056/NEJMoa074311 18354103

[B11] Litwin MS Gore JL Kwan L Brandeis JM Lee SP Withers HR Reiter RE Quality of life after surgery, external beam irradiation, or brachytherapy for early-stage prostate cancer Cancer 2007 109 2239 2247 10.1002/cncr.22676 17455209

[B12] Gore JL Gollapudi K Bergman J Kwan L Krupski TL Litwin MS Correlates of bother following treatment for clinically localized prostate cancer J Urol 2010 184 1309 1315 10.1016/j.juro.2010.06.012 20723914

[B13] Wiegner EA King CR Sexual function after stereotactic body radiotherapy for prostate cancer: results of a prospective clinical trial Int J Radiat Oncol Biol Phys 2010 78 442 448 10.1016/j.ijrobp.2009.07.1748 20137864

[B14] Reeve BB Potosky AL Willis GB Should function and bother be measured and reported separately for prostate cancer quality-of-life domains? Urology 2006 68 599 603 10.1016/j.urology.2006.03.037 16979720

[B15] Kimura M Banez LL Polascik TJ Bernal RM Gerber L Robertson CN Donatucci CF Moul JW Sexual bother and function after radical prostatectomy: predictors of sexual bother recovery in men despite persistent post-operative sexual dysfunction Andrology 2013 1 256 261 10.1111/j.2047-2927.2012.00036.x 23413138

[B16] Donohoe JE To what extent can response shift theory explain the variation in prostate cancer patients’ reactions to treatment side-effects? A review Qual Life Res 2011 20 161 167 10.1007/s11136-010-9745-y 20890663

[B17] Knight SJ Latini DM Hart SL Sadetsky N Kane CJ DuChane J Carroll PR Education predicts quality of life among men with prostate cancer cared for in the Department of Veterans Affairs: a longitudinal quality of life analysis from CaPSURE Cancer 2007 109 1769 1776 10.1002/cncr.22597 17380491

[B18] Aning JJ Wassersug RJ Goldenberg SL Patient preference and the impact of decision-making aids on prostate cancer treatment choices and post-intervention regret Curr Oncol 2012 19 S37 S44 23355792 10.3747/co.19.1287 PMC3553561

[B19] Gay HA Michalski JM Hamstra DA Wei JT Dunn RL Klein EA Sandler HM Saigal C Litwin M Kuban D Neoadjuvant androgen deprivation therapy leads to immediate impairment of vitality/hormonal and sexual quality of life: results of a msulticenter prospective study Urology 2013 82 1363 1369 10.1016/j.urology.2013.06.062 24139340 PMC3904352

[B20] Wei JT Dunn RL Litwin MS Sandler HM Sanda MG Development and validation of the expanded prostate cancer index composite (EPIC) for comprehensive assessment of health-related quality of life in men with prostate cancer Urology 2000 56 899 905 10.1016/S0090-4295(00)00858-X 11113727

[B21] Barry MJ Fowler FJ Jr O’Leary MP Bruskewitz RC Holtgrewe HL Mebust WK Cockett AT The American Urological Association symptom index for benign prostatic hyperplasia. The Measurement Committee of the American Urological Association J Urol 1992 148 1549 1557 discussion 1564 1279218 10.1016/s0022-5347(17)36966-5

[B22] Rosen RC Cappelleri JC Gendrano N 3rd The International Index of Erectile Function (IIEF): a state-of-the-science review Int J Impot Res 2002 14 226 244 10.1038/sj.ijir.3900857 12152111

[B23] Norman GR Sloan JA Wyrwich KW Interpretation of changes in health-related quality of life: the remarkable universality of half a standard deviation Med Care 2003 41 582 592 12719681 10.1097/01.MLR.0000062554.74615.4C

[B24] Crook J Fleshner N Roberts C Pond G Long-term urinary sequelae following 125iodine prostate brachytherapy J Urol 2008 179 141 145 discussion 146 17997424 10.1016/j.juro.2007.08.136

[B25] Salonia A Zanni G Gallina A Sacca A Sangalli M Naspro R Briganti A Farina E Roscigno M Dapozzo LF Baseline potency in candidates for bilateral nerve-sparing radical retropubic prostatectomy Eur Urol 2006 50 360 365 10.1016/j.eururo.2005.12.007 16413666

[B26] Keyes M Miller S Moravan V Pickles T Liu M Spadinger I Lapointe V Morris WJ Urinary symptom flare in 712 125I prostate brachytherapy patients: long-term follow-up Int J Radiat Oncol Biol Phys 2009 75 649 655 10.1016/j.ijrobp.2008.11.043 19211199

[B27] Cesaretti JA Stone NN Stock RG Urinary symptom flare following I-125 prostate brachytherapy Int J Radiat Oncol Biol Phys 2003 56 1085 1092 10.1016/S0360-3016(03)00210-4 12829146

[B28] Hamstra DA Conlon AS Daignault S Dunn RL Sandler HM Hembroff AL Zietman AL Kaplan I Ciezki J Kuban DA Multi-institutional prospective evaluation of bowel quality of life after prostate external beam radiation therapy identifies patient and treatment factors associated with patient-reported outcomes: the PROSTQA experience Int J Radiat Oncol Biol Phys 2013 86 546 553 10.1016/j.ijrobp.2013.01.036 23561651

[B29] Pontari M Giusto L New developments in the diagnosis and treatment of chronic prostatitis/chronic pelvic pain syndrome Curr Opin Urol 2013 23 565 569 10.1097/MOU.0b013e3283656a55 24080807

[B30] Pugh TJ Keyes M Barclay L Delaney A Krzywinski M Thomas D Novik K Yang C Agranovich A McKenzie M Sequence variant discovery in DNA repair genes from radiosensitive and radiotolerant prostate brachytherapy patients Clin Cancer Res 2009 15 5008 5016 10.1158/1078-0432.CCR-08-3357 19638463

[B31] Punnen S Cooperberg MR Sadetsky N Carroll PR Among potent men post radical prostatectomy, does the need for phosphodiesterase inhibitors have an impact on sexual bother scores? BJU Int 2012 109 1520 1524 10.1111/j.1464-410X.2011.10605.x 21999368 PMC3288727

[B32] Stensvold A Dahl AA Brennhovd B Smastuen MC Fossa SD Lilleby W Steinsvik A Axcrona K Smeland S Bother problems in prostate cancer patients after curative treatment Urol Oncol 2013 31 1067 1078 10.1016/j.urolonc.2011.12.020 22341412

